# Evaluation of the SOFA score as a tool to predict DCI-associated infarctions after spontaneous subarachnoid hemorrhage

**DOI:** 10.3389/fmed.2025.1580643

**Published:** 2025-06-23

**Authors:** Elena Kurz, Verena Fassl, Carolin Brockmann, Alicia Schulze, Darius Kalasauskas, Florian Ringel, Axel Neulen

**Affiliations:** ^1^Department of Neurosurgery, University Medical Center of the Johannes Gutenberg-University of Mainz, Mainz, Germany; ^2^Department of Neuroradiology, University Medical Center of the Johannes Gutenberg-University of Mainz, Mainz, Germany; ^3^Institute of Medical Biostatistics, Epidemiology and Informatics, University Medical Center of the Johannes Gutenberg-University of Mainz, Mainz, Germany

**Keywords:** subarachnoid hemorrhage, SAH, delayed cerebral ischemia, DCI, Sequential Organ Failure Assessment score, SOFA score, clinical

## Abstract

**Background:**

Delayed cerebral ischemia (DCI)-associated infarctions are a major complication after spontaneous subarachnoid hemorrhage (SAH). Besides cerebral pathophysiological effects, peripheral organ dysfunction has been associated with DCI. The Sequential Organ Failure Assessment (SOFA) score is used in intensive care medicine to monitor organ failure. The objective of our study was to compare the SOFA score obtained in the first 48 h post-SAH, Hunt & Hess (HH), and World Federation of Neurosurgical Societies (WFNS) scores in predicting DCI-associated infarctions and to identify the most robust parameters within the SOFA score.

**Methods:**

We retrospectively evaluated SOFA, H&H, and WFNS scores and DCI-associated infarctions in a cohort of 253 SAH patients.

**Results:**

The ROC analysis revealed an AUC of 0.65 for the SOFA score in predicting DCI-associated infarctions (H&H: 0.64, WFNS: 0.62). The threshold that maximized the sum of sensitivity and specificity was ≥7 points (sensitivity of 0.58, specificity of 0.68, PPV of 0.20, NPV of 0.92). A simplified score using only the three most robust parameters of the SOFA score, GCS, mean arterial pressure, and the Horovitz quotient, resulted in an AUC of 0.7.

**Conclusion:**

The SOFA score predicted the development of DCI-associated infarctions similar to the established H&H and WFNS scores. A simplified score combining the three most robust parameters of the SOFA score was at least equal to the established scores. Therefore, the SOFA score and our simplified score could be used as an additional tool to identify SAH patients at high risk for DCI-associated infarctions.

## Introduction

Spontaneous subarachnoid hemorrhage (SAH) accounts for approximately 5–10% of all strokes and has high rates of mortality and long-term disability (1).

In addition to early brain injury, which occurs as a direct consequence of intracranial bleeding and the associated transient global cerebral ischemia, delayed cerebral ischemia (DCI) is an important cause of secondary brain injury. In many patients, the course is further complicated by peripheral organ dysfunctions. In particular, neurogenic cardiomyopathy and neurogenic pulmonary edema occur in varying degrees in a large subgroup of SAH patients, as well as systemic inflammation, which can occur as a consequence of treatment-associated infections.

The pathophysiology of DCI is complex and not completely understood (2). In addition to cerebral pathophysiological effects such as vasospasms of large and small arteries, cortical spreading depressions, microthrombosis, cerebral inflammatory processes, and others, peripheral organ dysfunction has been associated with DCI in several studies (2–5). In particular, cardiac dysfunction—by reducing cerebral blood flow—and systemic inflammation—by triggering neuroimmunological effects—have been suggested to contribute to DCI (6–8).

Oral nimodipine, recommended in the weeks after SAH, has been demonstrated to reduce the rate of DCI-associated infarctions (9). Nevertheless, DCI still occurs in up to 30% of SAH patients and can lead to cerebral infarctions with consecutive functional deficits and a poor neurological outcome. Therapeutic options include inducing hypertension and endovascular salvage therapies in refractory cases. However, it is crucial to start these therapies before the manifestation of infarctions. Since early diagnosis of DCI is crucial, intensive monitoring of SAH patients at risk for DCI is a central element of therapy during the first 2–3 weeks after SAH (10).

It remains challenging to appropriately select patients at risk of DCI. It has been shown that the degree of EBI correlates with the risk of DCI. Accordingly, relatively high predictive values for predicting DCI have been shown for the Hunt & Hess (H&H) and the WFNS scores, both of which reflect the severity of EBI (11, 12).

These scores do not consider other organ dysfunctions. A score depicting inflammatory processes and dysfunction of other organ systems, in addition to brain injury, might be superior to these established scores in predicting DCI.

The Sequential Organ Failure Assessment (SOFA) score is routinely used in intensive care medicine to monitor organ dysfunction and is associated with mortality in sepsis (13–15). Other studies have shown that the score provides a holistic picture of inflammation and reflects organ dysfunction in critical illness (13, 14). The SOFA score is calculated by evaluating mean arterial blood pressure (MAP) and the need for vasopressors as measures of cardiac function, the Glasgow Coma Scale (GCS) as a measure of brain injury, the Horovitz quotient as a measure of the oxygenation capacity of the lung, and serum bilirubin, platelet counts, and creatinine levels (14, 16). Lambden et al. (14) reported that an increase of 2 points in the SOFA score was associated with a mortality rate of approximately 10% in ICU patients with infections, and it has been shown to have better prognostic accuracy for the prediction of mortality in case of critical illness than other routinely used scores. The SOFA score has also been correlated with mortality and outcome after SAH (17, 18).

It remains unclear, however, whether the SOFA score could also be used to predict DCI and to select SAH patients at high risk of DCI and DCI-associated infarctions.

Therefore, our study aimed to investigate whether the SOFA predicts the occurrence of DCI-associated infarctions and to compare it to the established H&H and WFNS scores. We further set out to analyze the ability of the different parameters contributing to the SOFA score to predict DCI-associated infarctions and to compose a simplified new score based on the most robust parameters.

## Materials and methods

### Study design and ethical approval

This study was designed as a retrospective observational cohort study and was approved by the Ethics Committee of the Rhineland-Palatinate Chamber of Physicians. The study was conducted in accordance with the Declaration of Helsinki and its later amendments. Due to the retrospective nature of the study and the use of anonymized data, informed consent was waived.

### Setting and participants

We retrospectively included all adult patients (≥18 years) admitted to the neurosurgical intensive care unit (ICU) of our tertiary care center for aneurysmal subarachnoid hemorrhage (SAH) over 10 years (January 2011–December 2021). Clinical management followed standardized departmental protocols throughout the study period (19, 20).

Patients were eligible for inclusion if the following criteria were met:

1. Complete data were available to calculate the Sequential Organ Failure Assessment (SOFA) score on days 1 and 2 following SAH.
2. A cranial CT or MRI scan was obtained between days 14 and 28 post-SAH to evaluate for delayed cerebral ischemia (DCI)-related infarctions.

### Data sources and measurement

SOFA score parameters were extracted from electronic medical records. For each patient, the highest SOFA score recorded within the first 48 h after ICU admission was used for analysis. Cranial imaging performed between days 14 and 28 post-SAH was independently reviewed by a board-certified neuroradiologist blinded to clinical and outcome data. Infarctions were classified as DCI-associated if no alternative etiology (e.g., procedural complications, rebleeding) could be identified. Imaging data were reviewed using Sectra Workstation IDS7 (Version 17.1.10, Sectra AB, Linköping, Sweden).

The following baseline characteristics were documented as follows: age, sex, comorbidities, aneurysm location, treatment modality (clipping or endovascular treatment), Hunt and Hess (H&H) grade, Fisher’s score, and World Federation of Neurosurgical Societies (WFNS) grade. Functional outcome at 6 months was assessed using the modified Rankin Scale (mRS) score.

### Bias and study size

To minimize misclassification bias, imaging interpretation was performed independently and in a blinded manner. No sample size calculation was performed due to the retrospective design; all eligible patients during the study period were included.

### Statistical analysis

Data were described using absolute and relative frequencies for categorical variables and mean and standard deviation (SD) for continuous variables. Logistic regression analysis was performed to assess associations between clinical variables, the occurrence of cerebral infarctions, and 6-month functional outcomes. Differences between predefined score-based subgroups regarding the incidence of DCI were evaluated using the chi-squared test.

As a requirement for continuous independent variables in the logistic regression, all scores fulfilled the assumption of linearity of the logit, while the six variables contributing to the SOFA are categorical.

Each SOFA component was individually assessed for its discriminatory power in predicting DCI using the area under the receiver operating characteristic curve (AUC). The three variables with the highest AUCs were selected to develop simplified models, which included three, two, and one predictor variable(s).

To identify the most predictive and parsimonious models, all possible combinations of SOFA variables (in sets of three, two, and one) were evaluated using the Akaike information criterion (AIC). The models with the lowest AICs in each set were selected as candidate simplified scores. The combinations, based on the individual predictors with the highest AUC, also showed superior performance in terms of model fit.

The receiver operating characteristic (ROC) curve analysis was conducted for each model, and the predictive performance was quantified using AUC values. Comparisons between the newly developed models and the existing scoring systems were performed using the DeLong test. Optimal thresholds for DCI prediction were determined using the Youden index. All statistical analyses were conducted using R statistical software (version 4.4.1; R Core Team, 2021) (23).

## Results

**Figure 1:**
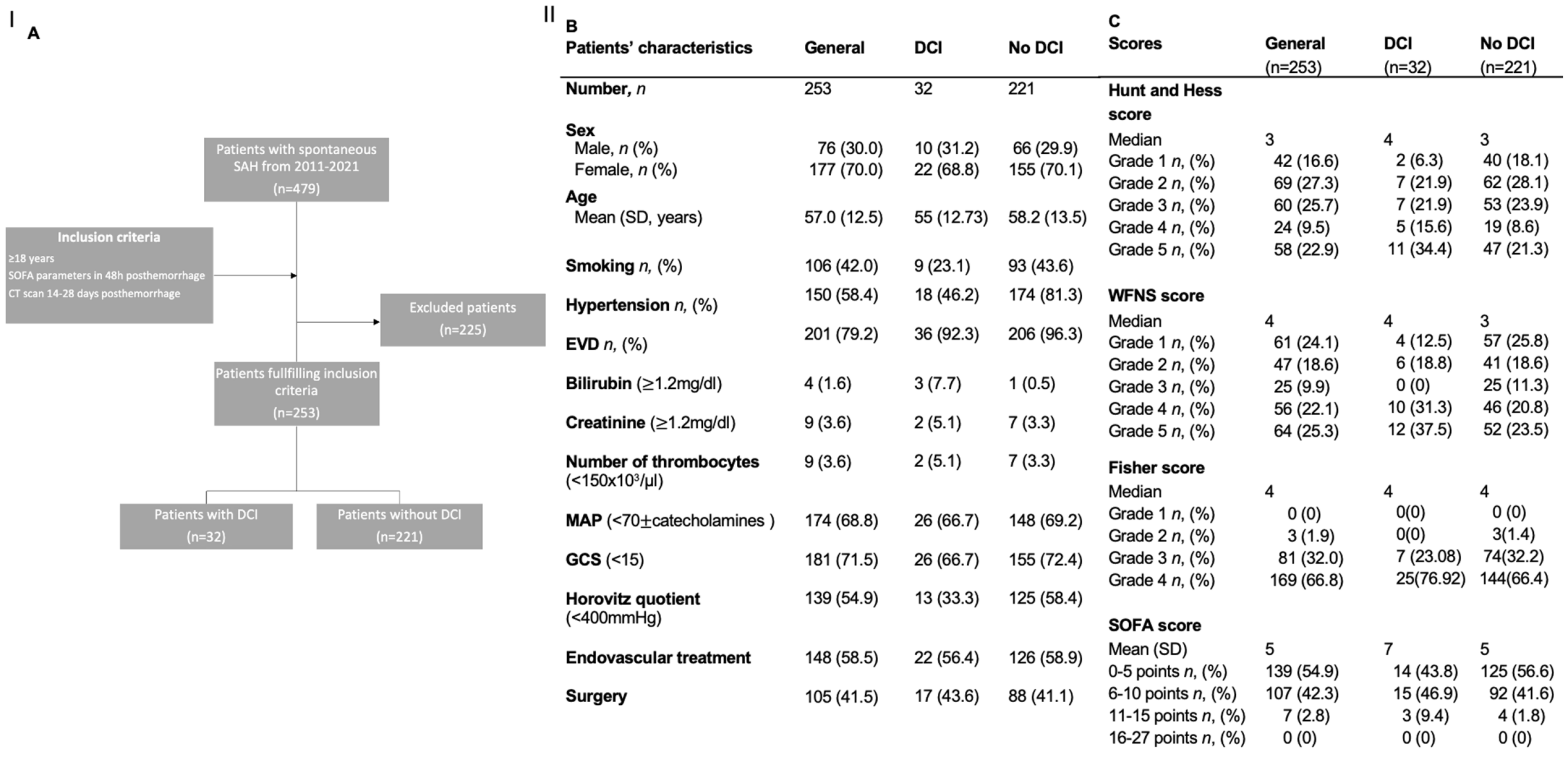
**(A)** Flowchart illustrating the selection process for the inclusion of patients. **(B,C)** Demographics and scores of the study population and for the subgroups of patients with and without DCI. AUC: area under the curve; DCI: delayed cerebral infarctions; EVD: external ventricular drainage; MCA: middle cerebral artery; ICA: internal cerebral artery; SD: standard deviation; SOFA: Sequential Organ Failure Assessment score; WFNS: World Federation of Neurosurgical Societies score.

We screened 479 patients with spontaneous SAH treated in the Department of Neurosurgery of the University Hospital in Mainz, Germany, between January 2011 and December 2021. A total of 253 patients fulfilled the inclusion criteria (Figure 1). A total of 69.9% (*n* = 177) of the included patients were female, and 30.04% (*n* = 76) of the patients were male. The mean age was 57.0 years (SD = 12.5). Aneurysms of the anterior communicating artery were the most frequent source of SAH (*n* = 70, 27.2%), followed by aneurysms of the MCA (*n* = 49, 19.4%) and pCom aneurysms (*n* = 30, 11.86%). A total of 49 patients (19.4%) had multiple aneurysms. Additionally, 42% (*n* = 106) of the included patients were smokers, and 60% (*n* = 152) had arterial hypertension. The median grade of the HH score was 3, the median of the WFNS score was 3 as well, and the median Fisher’s score was 4. The median SOFA score was 5. Nearly 80% of the patients (*n* = 201) developed transient hydrocephalus. A total of 19.8% (*n* = 50) patients required continuous cerebrospinal fluid (CSF) drainage and were implanted with a ventriculoperitoneal shunt. Vasospasm was detected on digital subtraction angiography in 36.4% of patients (*n* = 92). Of these, 24.0% (*n* = 60) of patients underwent vasospasm therapy. Moreover, patients (12.6% of the whole cohort) developed DCI-associated infarctions. The mean mRS at 6 months was 3.1 (SD = 1.9). More details are shown in Table 1.

**Table 1:** The sequential organ failure assessment score – parameters and grading.

Points	Creatinine (mg/dL)	Bilirubin (mg/dL)	Number of thrombocytes (×10^3^/μL)	Horovitz quotient (PaO_2_/FiO_2_, mmHg)	MAP	GCS
0	<1.2	<1.2	≥150	≥400	≥70 mmHg	15
1	1.2–1.9	1.2–1.9	<150	<400	<70 mmHg	14-13
2	2.0–3.4	2.0–5.9	<100	<300	Dopamine (≤5 μg/kg/min) OR dobutamine	12-10
3	3.5–4.9	6.0–11.9	<50	<200 + ventilation	Dopamine (>5 μg/kg/min) or epinephrine (≤0.1 μg/kg/min) or norepinephrine (≤0.1 μg/kg/min)	9-6
4	>5	>12.0	<20	<100 + ventilation	Dopamine (>15 μg/kg/min) or epinephrine (>0.1 μg/kg/min) OR norepinephrine (>0.1 μg/kg/min)	<6

### DCI-associated infarctions

In 32 (12.6%) cases, DCI-associated infarctions were detected.

Our cohort was divided into two groups: patients who experienced a DCI-associated infarction (*n* = 32) and patients without DCI-associated infarction (*n* = 221). The two groups did not differ significantly in terms of sex distribution, age, or risk factors for hypertension and smoking.

Concerning the evaluated scores, the two groups exhibited only marginal differences.

The development of hydrocephalus, the number of aneurysms, and the outcome of both groups in comparison, as well as more details, are shown in Table 1.

We correlated the evaluated parameters with the development of delayed cerebral infarctions and analyzed their predictive value.

In the univariate regression analysis, SOFA (*p* = 0.005, OR = 1.22, 95% CI = 1.07–1.41), WFNS (*p* = 0.03, OR = 1.34, 95% CI = 1.04–1.77), HH (*p* = 0.01, OR = 1.43, 95% CI = 1.09–1.91), and multiple aneurysms (*p* = 0.04, OR = 0.42, 95% CI = 1.12–1.94) were significantly correlated with the development of infarctions after SAH. The need for an Extraventricular Drainage (EVD) showed a trend toward a correlation with the development of infarctions (*p* = 0.05, OR = 4.12, 95% CI = 0.96–17.7).

We included the abovementioned parameters in the multivariate analysis and found that only the SOFA score (*p* < 0.0001, OR = 1.31, 95% CI = 1.13–1.53) and the HH score (*p* = 0.03, OR = 1.5, 95% CI = 1.05–2.05) remained independent significant predictors of delayed cerebral infarctions.

The scores (HH, WFSN, and SOFA score), which correlated with delayed cerebral infarctions in univariate analysis, were further analyzed.

The Hunt and Hess score’s threshold comes with a significantly higher risk for delayed cerebral infarctions if exceeded, which was graded 4 or higher. Patients with an HH lower than 4 developed 8% infarctions, whereas infarctions were found in 17% of patients with an HH from 4 to 5 (*p* = 0.023). The Youden index was 0.22. The positive predictive value was 0.2, the negative predictive value was 0.91, the sensitivity was 0.51, and the specificity was 0.7.

The evaluation of the predictive value of the HH score for delayed cerebral infarctions is shown in Figure I 2B. The ROC curve shows an AUC of 0.64 (95% CI = 0.54–0.74) (Figure I 2B), and the AIC was 184.53.

**Figure 2:**
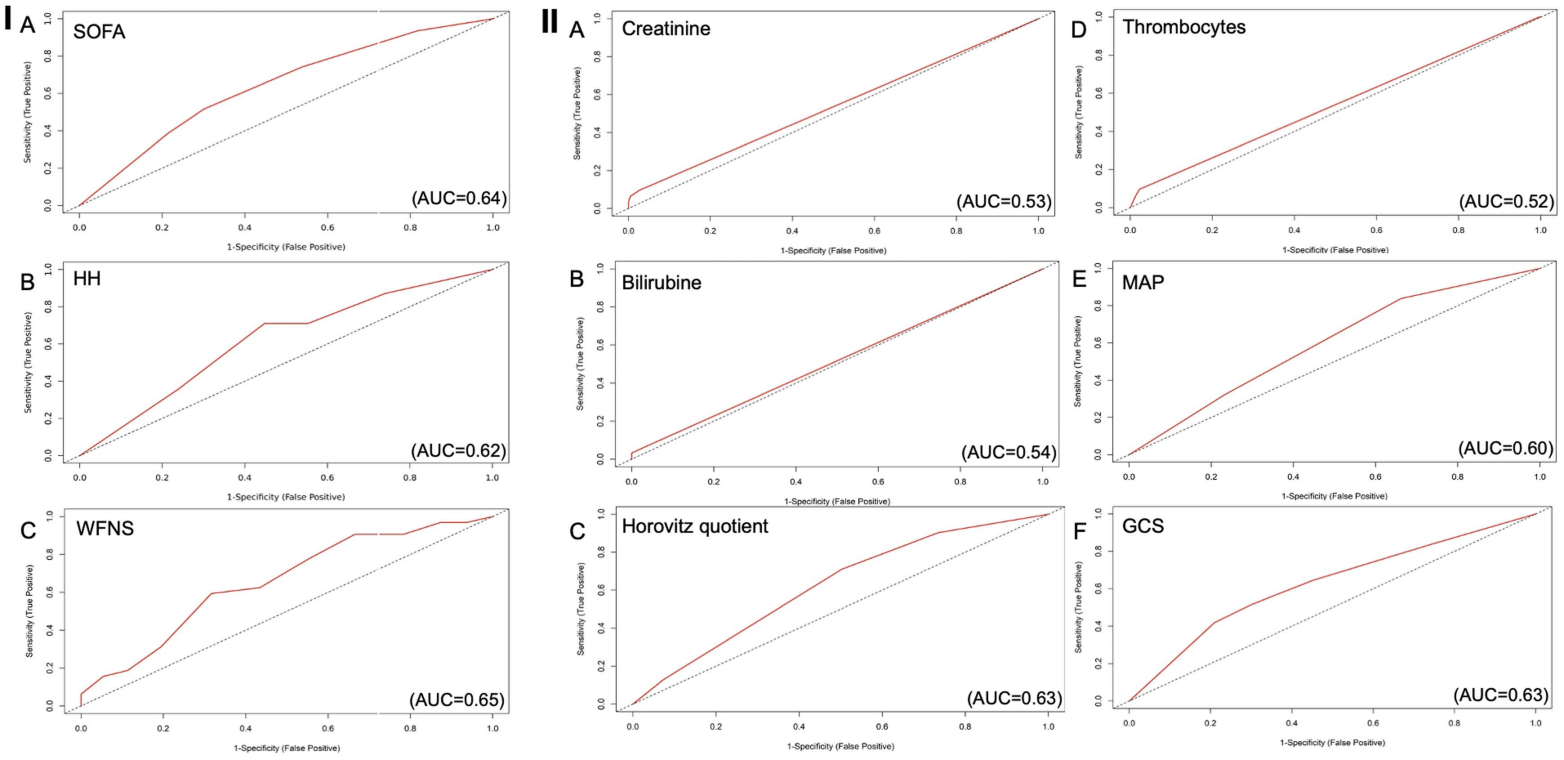
ROC curves for every parameter of the SOFA score as predictor for delayed cerebral infarctions, AUC 0.66 (C197.5%=0.56-0.75). **(A)** Creatinine as predictive value for DCI (AUC=0.53), **(B)** Bilirubin as predictive value for DCI (AUC=0.54), **(C)** Horovitz quotient as predictive value for DCI (AUC=0.63), **(D)** Number of thrombocytes as predictive value for DCI (AUC=0.52), **(E)** MAP as predictive value for DCI, (AUC=0.60) **(F)** GCS as predictive value for DCI (AUC=0.63). AUC: Area under the curve; DCI: Delayed Cerebral Infarctions; GCS: Glasgow Coma Scale; MAP: Mean Arterial Pressure.

The threshold for the WFNS score indicating a higher risk of delayed cerebral infarctions was grade 4 or higher. Patients with a WFNS score of 1–3 developed a delayed cerebral infarction in 8% of cases, whereas patients with a WFNS score of 4 or higher were affected in 18% (*p* = 0.0035). The positive predictive value was 0.18, and the negative predictive value was 0.93. The sensitivity was 0.71 and the specificity was 0.55, with the Youden index of 0.26.

The ROC curve depicting the predictive value of the WFNS score for delayed cerebral infarctions is shown in Figure I 2C. The AUC was 0.62 (95% CI = 0.52–0.72) (Figure I 2B) and the AIC was 186.19.

The ROC curve evaluating the predictive value of the SOFA score for delayed cerebral infarctions is shown in Figure I 2A. The AUC was 0.65 (95% CI = 0.55–0.75), and the AIC was 182.94. The cutoff lies at 7 points, with a higher risk if this value is equal to or exceeded.

The calculated threshold for the SOFA score, above which there was a higher risk of infarctions, was 7 points. Patients with a score of 7 or lower were affected by delayed cerebral infarctions in only 8% of cases, which was significantly less than the 24% of patients with a SOFA score of 7 or higher (*p* = 0.0027). The Youden index was 0.26, with the highest predictive value for delayed cerebral infarctions among all analyzed scores. The positive predictive value was 0.20, the negative predictive value was 0.92, sensitivity was 0.58, and specificity was 0.68 (Figure I 2A).

In comparison, the ROC analysis for predicting DCI using the WFNS score resulted in an AUC of 0.62. The HH score achieved an AUC of 0.64, while the FS score achieved an AUC of 0.56.

In the next step, we checked every parameter of the SOFA score for its predictive value. The most valuable parameters analyzed on their own with regard to their discriminatory value (AUC) were the GCS (AUC = 0.63, sensitivity = 0.42, specificity = 0.79, PPV = 0.22, NPV = 0.91) with the Youden index of 0.21 and the Horovitz quotient (AUC = 0.63, sensitivity = 0.71, specificity = 0.50, PPV = 0.17, NPV = 0.92) with the Youden index of 0.21. The last parameter with the third highest AUC was the MAP (AUC = 0.60, sensitivity = 0.84, specificity = 0.34, PPV = 0.15, NPV = 0.94) (Figures II 3A–F).

**Figure 3:**
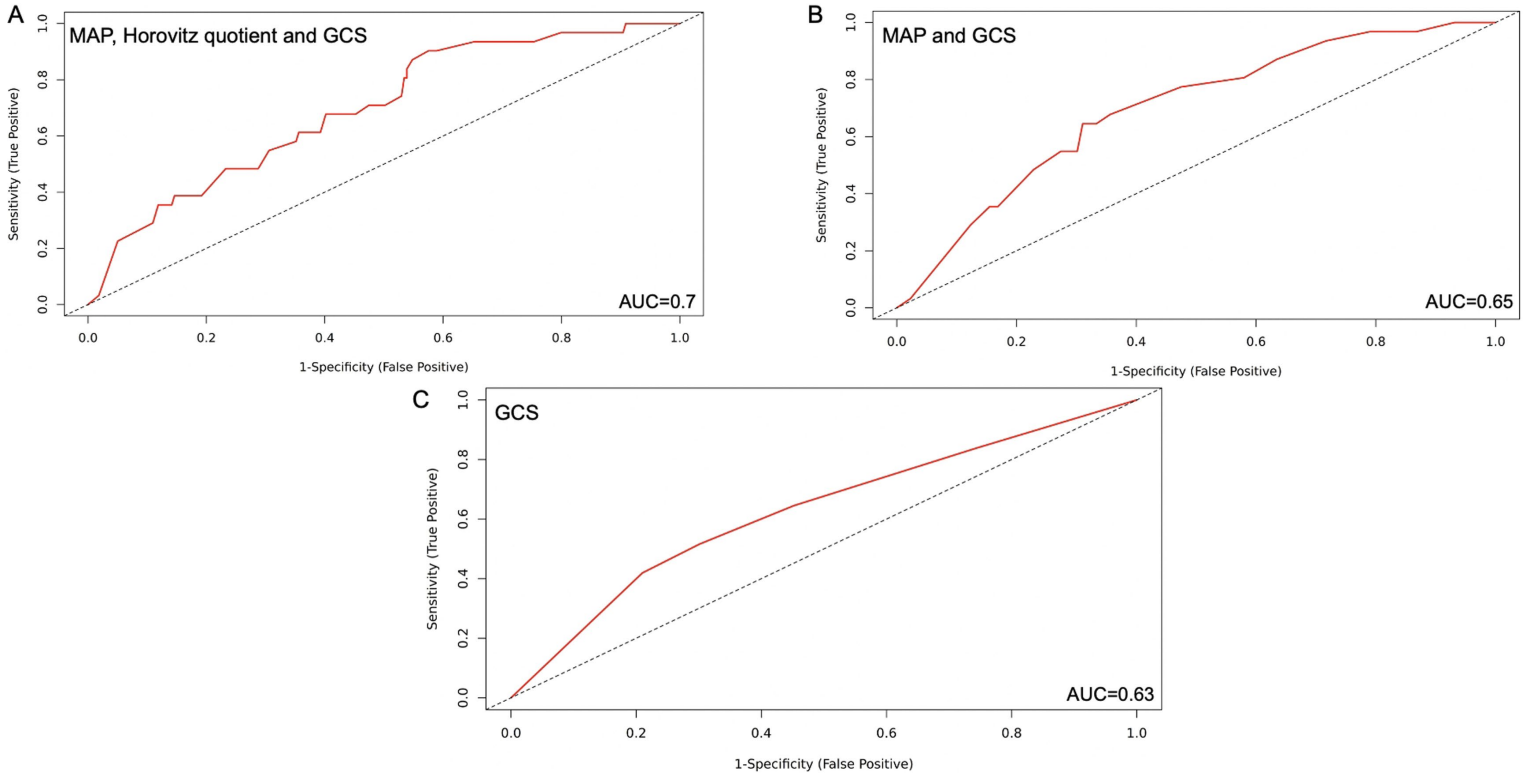
Simplified SOFA score with 1,2 and 3 parameters respectively with the highest predictive value for DCI-associated infarctions after SAH. **(A)** MAP, Horovitz quotient and GCS included, AUC=0.70 **(B)** MAP and GCS, AUC=0.65 **(C)** GCS alone, AUC=0.63. AUC: Area under the curve; GCS: Glasgow Coma Scale; MAP: Mean Arterial Pressure; SAH: Subarachnoidal hemorrhage; SOFA; Sequential Organ Failure Assessment Score.

Creatinine (AUC = 0.53), bilirubin (AUC = 0.54), and the number of thrombocytes (AUC = 0.52) achieved lower AUC values when used independently of the score.

We evaluated all parameters of the SOFA score in combination for their reliability for the prediction of DCI to create a simple but, at the same time, reliable score (Figures 3A–C).

In line with the model selection strategy, the simplified scores incorporating the SOFA components with the highest individual AUC values demonstrated superior predictive performance for DCI, as indicated by the lowest AIC values across all tested combinations.

Following that, the most clinically reliable modified score, demonstrating a solid predictive value, was the combination of GCS, Horovitz quotient, and MAP. When each parameter was scored with 0–4 points, as in the original score, patients with a total score between 0 and 6 points developed DCI in 8.2% of cases, while those with a score between 7 and 12 points developed DCI in 21.7% of cases (*p* = 0.004). The AIC was 192.93, with an AUC of 0.70. The sensitivity was 0.84, and the specificity was 0.49. The PPV reached a value of 0.19, and the NPV was 0.96. The Youden index was 0.33 (Figure 3A).

The AUC of the simplified three-parameter score did not differ significantly from those of the SOFA score, the HH score, and the WFNS score (*p* = 0.3, *p* = 0.2, and *p* = 0.1, respectively).

The combination of two parameters with the highest AUC and clinical reliability was the GCS and the Horovitz quotient. When every parameter was scored with 0–4 points as in the original score, we found 8.2% DCI in the group of patients with a score from 0 to 3 and in the group of patients with a score from 4 to 8 points, with 21.9% DCI (*p* = 0.004). The AIC was 191.12, and the AUC was 0.7. The sensitivity of this model was 0.65; the specificity was 0.7, the PPV was 0.23, the NPV was 0.93, and the Youden index was 0.33 (Figure 3B).

The AUC of the simplified two-parameter score was not significantly different from those of the SOFA, HH, and WFNS scores (*p* = 0.4, *p* = 0.2, and *p* = 0.2, respectively). Similarly, a comparison with the three-parameter score revealed no statistically significant difference (*p* = 0.9).

As a predictive factor alone, the GCS had an AIC of 190.9 with an AUC of 0.63. Patients with a score of 0–3 had a risk of DCI of 8.6%, and those with a score of 4 had a risk of 24.2% (*p* = 0.006). In this case, the sensitivity was 0.42, the specificity was 0.79, the PPV was 0.22, and the NPV was 0.92. The Youden index was 0.21 (Figure 3C).

The AUC of the GCS alone was not inferior to those of the established scores (SOFA, HH, and WFNS), with *p*-values of 0.7, 0.8, and 0.9, respectively. In the DeLong test, there was no significant superior predictive performance of both the three-parameter and two-parameter scores compared to the GCS alone (*p* = 0.1 for both comparisons).

## Discussion

The present study, to the best of our knowledge, shows for the first time that the highest SOFA score determined during the first 48 h after SAH onset predicts the occurrence of DCI-associated infarctions. We also demonstrate that a simplified score derived from the SOFA score, including only the subcategories for consciousness, cardiac, and respiratory function, performs similarly to the SOFA, HH, and WFNS scores. This simplified score is simple, widely used, and could be easily implemented into clinical practice.

DCI and DCI-associated infarctions are a central aspect of deteriorating neurological outcomes in SAH patients. Their occurrence underlies a complex pathophysiology. Several cerebral pathophysiological processes have been identified, including vasospasms of large vessels and microvessels, microthrombosis, cortical spreading depressions, and cerebral inflammatory processes (2–4, 10, 21, 22). Furthermore, peripheral organ dysfunction has been reported to contribute to DCI, particularly through reduced cardiac output in the context of neurogenic cardiomyopathy (23, 24).

The SOFA score is used to monitor organ dysfunction and was also shown to correlate with systemic inflammation in ICU patients (13, 15, 16).

There are several reasons why it is plausible that high SOFA scores are associated with an increased risk of DCI-associated infarctions in SAH patients.

First, the GCS component of the SOFA score assesses the patient’s level of consciousness, which correlates with the extent of primary brain injury following SAH. The established HH and WFNS scores similarly evaluate the extent of primary brain injury. The extent of primary brain injury has been shown to correlate with the risk of DCI and DCI-associated infarctions. Consequently, patients with high scores in this category are at higher risk of DCI and DCI-associated infarctions.

Second, systemic inflammation has been shown to trigger cerebral inflammation by promoting blood-brain barrier disruption and microglia activation (25), presumably contributing to the development of DCI and DCI-associated infarctions (6–8). A high SOFA score could be due to systemic inflammation, potentially enhancing the risk of DCI and DCI-associated infarctions.

Third, cerebral perfusion in the acute phase after SAH cannot be solely determined by cerebral vascular effects, but cardiac function plays a role (7, 26, 27). Neurogenic cardiomyopathy occurs as a result of excessive catecholamine release following the consequence of the bleeding event. It can occur in several degrees, from abnormal ECG findings to signs of cardiac insufficiency and pulmonary edema (23, 28).

Severe neurogenic cardiomyopathy has been frequently associated with low blood pressure and the need for vasopressors. Additionally, severe cardiac insufficiency can trigger pulmonary edema in some patients. In short, neurogenic cardiomyopathy can lead to higher scores in the cardiac and respiratory categories of the SOFA score. Our finding indicates that the most robust categories of the SOFA score were the MAP and Horovitz quotient, alongside the GCS, which highlights the important role of these pathophysiological mechanisms.

The occurrence of DCI and DCI-associated infarctions is a major factor contributing to unfavorable neurological outcomes after SAH and has been reported to increase treatment time in the ICU and overall treatment costs significantly (29). Close monitoring of SAH patients to detect DCI early before the manifestation of infarctions, therefore, presents a central element of therapy. Early identification of patients at high risk for DCI or those with low risk of DCI, respectively, would be highly advantageous to optimize resource input. Monitoring could be de-escalated in patients with low DCI risk and focused on high-risk patients. However, it remains challenging to identify SAH patients with a high risk of DCI and DCI-associated infarctions early after hospital admission, although many approaches using different scores and clinical findings have been published (11, 12).

Several risk factors for the evaluation of the risk of DCI have been identified. Importantly, scores representing the degree of primary brain injury, such as HH and WFNS scores, have been shown to correlate with the risk of DCI and DCI-associated infarctions. Furthermore, other risk factors, such as age, aneurysm location, female sex, pre-morbid diabetes mellitus, and smoking, have been reported (30). However, these parameters are not classified in a scoring system, and their predictive value has been discussed as contradictory. There is, therefore, no consensus on which of these parameters should be used for prediction and how they should be graded (30). Of the non-invasive technical procedures, transcranial Doppler sonography has been shown to correlate with angiographic vasospasm. However, the value of vasospasm detection alone has been questioned in recent years because only a subgroup of patients with angiographic vasospasms develops DCI (26).

In our cohort, the predictive values of the established HH and WFNS scores and the SOFA score were similar, as along with their AUC in the ROC analysis. The simplified score derived from the SOFA score, focusing only on GCS and the cardiac and respiratory subcategories, performed at least as well as the established scores. The reduced 3-parameter score performs well in predicting delayed cerebral ischemia (DCI) following subarachnoid hemorrhage (SAH), likely due to its targeted focus on organ systems that are pathophysiologically relevant in the context of SAH. By concentrating on parameters with the highest AUC and a direct link to SAH-related complications, such as respiratory, cardiovascular, and neurological dysfunction, the simplified score improves both sensitivity and specificity for DCI prediction. In contrast, the inclusion of organ systems less commonly involved in the acute course of SAH, such as hepatic or hematologic parameters, may introduce statistical noise and obscure clinically meaningful patterns.

Although the modified models show a slight increase in AIC, the area under the ROC curve (AUC) remains comparable or even improved. This finding suggests that, despite a minor reduction in overall model fit, the ability of the models to discriminate between outcomes is preserved. Since AUC reflects the discriminative power of a model and is independent of model complexity, the findings indicate that the predictive performance of the simplified scores remains robust.

The SOFA score is routinely used in intensive care medicine to monitor the degree of organ dysfunction. It is easy to calculate, as all parameters are usually collected. Therefore, both the SOFA score and our simplified score, which focus solely on GCS and the cardiac and respiratory subcategories, could be easily integrated into clinical routine. Considering their integration, the SOFA score and our simplified score could be reasonably used as additional prediction tools for DCI-associated infarctions after SAH.

## Conclusion

The SOFA score measures the degree of peripheral organ dysfunction and was reported to correlate with systemic inflammation, which regularly accompanies SAH and has been believed to contribute to the development of DCI. We applied the highest SOFA score determined within the first 48 h after hospital admission to predict DCI-associated infarctions and compared the predictive value to the HH and WFNS scores. We found that the SOFA score was similarly effective at predicting DCI-associated infarctions. Consciousness and respiratory and cardiac function were the most robust subcategories of the SOFA score. A simplified score based solely on these parameters performed well and showed similar predictive ability to the HH and WFNS scores for DCI-associated infarctions. The SOFA score, which is routinely calculated daily in many intensive care units, as well as our simplified score, could therefore be used as additional instruments to early identify SAH patients at high or low risk of DCI-associated infarctions.

## Limitations

There are some limitations to this study. First, the retrospective character of the study can cause a selection bias, and our study is a single-center evaluation. The generalizability of the results is limited, as the results are based on a cohort of patients treated in the same environment and with the same treatment protocol. Additionally, the patient cohort was quite heterogeneous, as it included all grades of hemorrhages, treatments for vasospasms, and different types of endovascular surgical treatment of aneurysms were included.

We developed the modified SOFA score to predict DCI-associated infarctions based on the presented patient cohort. External validation on an independent patient collective has not yet been performed, which is our future goal.

## Data Availability

The raw data supporting the conclusions of this article will be made available by the authors, without undue reservation.
