# Correction: Evaluation of the SOFA score as a tool to predict DCI-associated infarctions after spontaneous subarachnoid hemorrhage

**DOI:** 10.3389/fmed.2025.1661506

**Published:** 2025-09-19

**Authors:** Elena Kurz, Verena Fassl, Carolin Brockmann, Alicia Schulze, Darius Kalasauskas, Florian Ringel, Axel Neulen

**Affiliations:** ^1^Department of Neurosurgery, University Medical Center of the Johannes Gutenberg-University of Mainz, Mainz, Germany; ^2^Department of Neuroradiology, University Medical Center of the Johannes Gutenberg-University of Mainz, Mainz, Germany; ^3^Institute of Medical Biostatistics, Epidemiology and Informatics, University Medical Center of the Johannes Gutenberg-University of Mainz, Mainz, Germany

**Keywords:** subarachnoid hemorrhage, SAH, delayed cerebral ischemia, DCI, Sequential Organ Failure Assessment score, SOFA score, clinical

There was a mistake in Figure 1 as published. There are incorrect values in section C of the figure. The corrected Figure 1 appears below.

**Figure 1:**
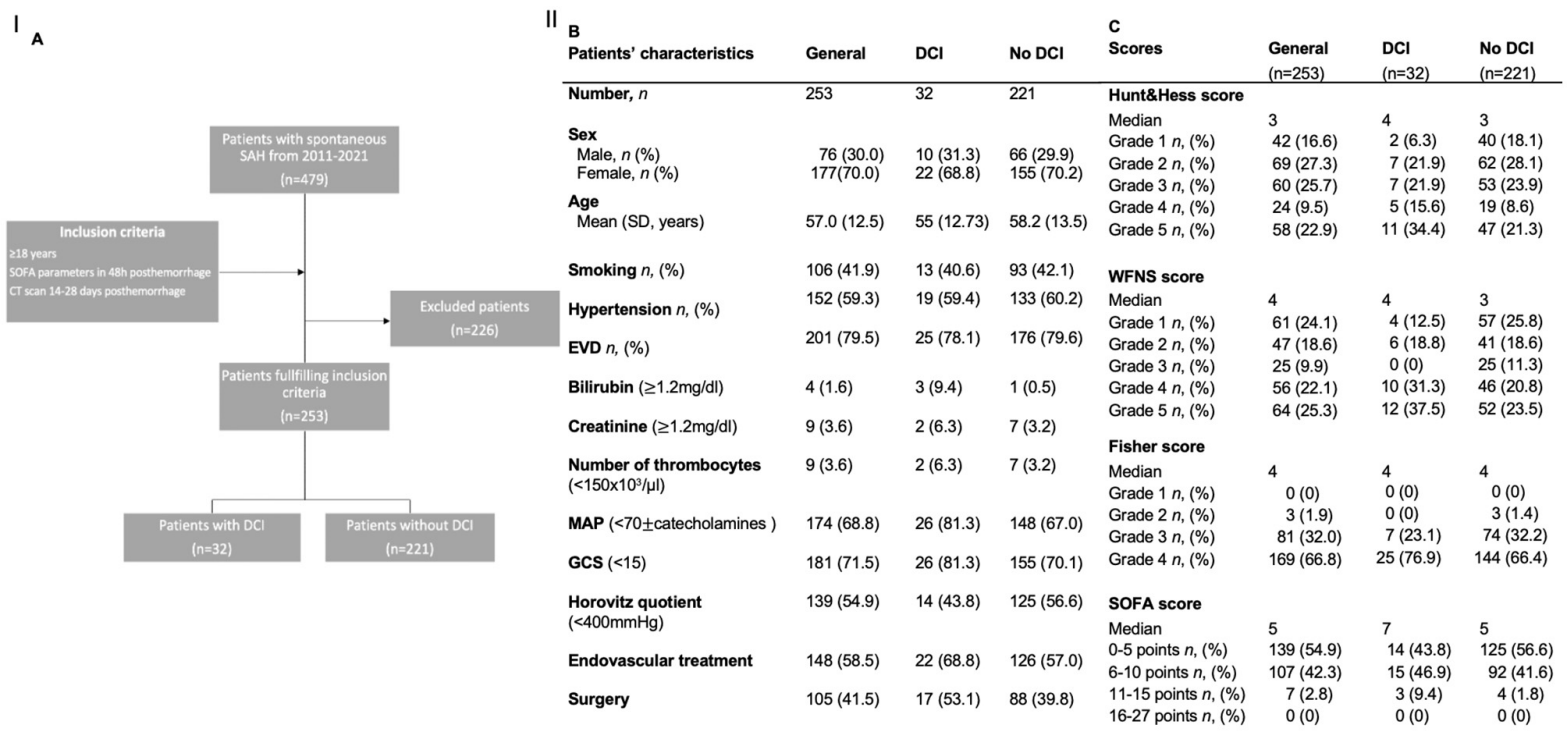
**(A)** Flowchart illustrating the selection process for the inclusion of patients. **(B, C)** Demographics and scores of the study population and for the subgroups of patients with and without DCI. AUC: area under the curve; DCI: delayed cerebral infarctions; EVD: external ventricular drainage; MCA: middle cerebral artery; ICA: internal cerebral artery; SD: standard deviation; SOFA: Sequential Organ Failure Assessment score; WFNS: World Federation of Neurosurgical Societies score.

The original version of this article has been updated.

